# Public Response, Anxiety and Behaviour during the First Wave of COVID-19 Pandemic in Saudi Arabia

**DOI:** 10.3390/ijerph18094628

**Published:** 2021-04-27

**Authors:** Amani Salem Alqahtani, Meshael Mohammed Alrasheed, Ada Mohammed Alqunaibet

**Affiliations:** 1 Saudi Food and Drug Authority, Riyadh 13513, Saudi Arabia; mmrasheed@sfda.gov.sa; 2 Saudi Center for Disease Prevention and Control, Riyadh 13354, Saudi Arabia; Alqunaibetm@moh.gov.sa

**Keywords:** COVID-19, pandemic, public response, Saudi Arabia, practices, preventive measures

## Abstract

This study aims to investigate public response attitude, anxiety, practices and trust in the authorities’ mitigation plan during the first wave of COVID-19 pandemic. A national cross sectional phone survey was conducted among Saudi residents aged 16 years and above. A total of 90,421 (45.2%) individuals participated in the study. Of those, the overall rate of COVID-19 correct knowledge was 82% (mean: 9.84); social media was the most reported source of knowledge. Younger age, low levels of education and foreign residents were associated with poor knowledge. Overall, 49.5% scored 5 or more on the GAD-7 test, indicating anxiety symptoms, 19.2% of them scored 10 and above, suggesting moderate to severe anxiety. Majority of participants (>78%) trusted and supported the interventions implemented by the government to control COVID-19. Social distancing practices among participants was as following, 72.5% stayed at home and avoid going out for nonessential business and 49.5% avoided attending social events and family gatherings. Trust in authorities, being anxious, worry and levels of knowledge about the disease, were the most common factors affecting adoption of the recommended practices. Continuous evaluation of public response about COVID-19, and the effectiveness of protective measures is essential to better inform policy-makers and identify ways of encouraging behaviour change among public during pandemic.

## 1. Introduction

Coronavirus disease 2019 (COVID-19) is an emerging respiratory infection caused by severe acute respiratory syndrome coronavirus 2 (SARS-CoV-2) [1]. Since its first discovery in China in December 2019, COVID-19 has widley spread and affected more than 85,403 people and spread to more than 54 countries around the world as of 29 February 2020. In Saudi Arabia, the first case was reported on 3 March 2020, and since then, the number of confirmed cases has reached 35,432 within the first 8 weeks [2]. Due to its contagiousness and global spread, on 11 March 2020, the World Health Organization (WHO) characterised the disease as a global pandemic [3].

**Figure 1:**
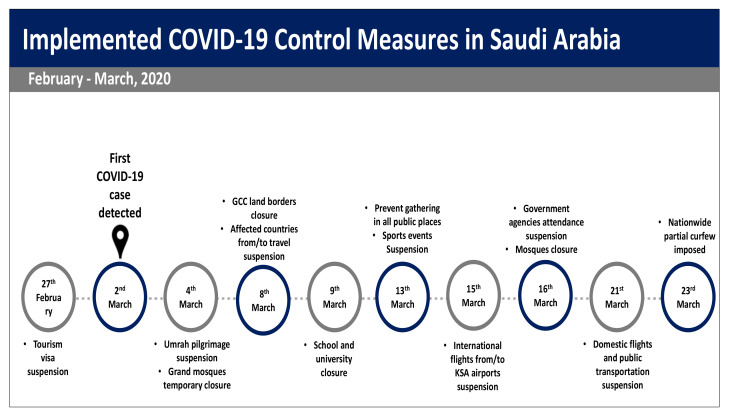
Timeline of Saudi Arabia response to compact COVID-19 spread in the first 4 weeks.

Saudi Arabia has adopted various national response strategies aiming to control the pandemic curve, starting with travel suspension, shutdown of schools, limiting workspace access and curfew (Figure 1). An additional implemented containment strategy is focussing on tracing, treating and isolating infected peoples as well as educating the public about personal protective measures, including hand hygiene, cough etiquette and social distancing to reduce the risk of transmission [4]. Studies have found that the wide coverage of personal protective measures among the population helped delay an influenza pandemic and decreased the infection rate, possibly reducing transmission and sufficiently containing the pandemic [5,6,7]. However, encouraging the public to adhere to protective measures could be challenging. The adoption of these measures is affected mostly by individuals’ knowledge, attitudes and perception of the disease [8]. The previous experience of Middle East respiratory syndrome coronavirus (MERS-CoV) outbreak in Saudi Arabia showed that although Saudi Arabia was the epicentre of MERS-CoV, majority of people were not concerned and thought the risk of the disease was only media propaganda. Thus, many people lacked accurate information on MERS-CoV transmission and prevention, and were not fully compliant with the preventive measures [8,9,10].

While almost all countries around the globe have applied unique measures that have not been undertaken previously, no study have investigated whether trust on authorities control plan and actions can affect public behaviour and anxiety level during COVID pandemic or not. Understanding the public’s response and behaviour is vital in designing policies, control strategic plan and preventive measures. Moreover, it can also inform policy-makers and identify ways of encouraging behaviour change among public during the early stages of the pandemic. To this end, this study is intended to understand the public concerns and behaviours at the beginning of COVID-19 pandemic in Saudi Arabia. 

## 2. Materials and Methods

### 2.1. Study Design

This study is a national cross-sectional survey conducted among a sample of Saudi residents aged 16 years and above. The study was conducted between 17 and 18 March 2020. The survey was developed using a web-based questionnaire, integrated with a recruitment system and secure database to store the collected data. Data were collected from 13 administrative regions in Saudi Arabia, including Al-Riyadh, Makkah, Al-Madinah, Al-Qaseem, Eastern Region, Aseer, Tabouk, Hail, Northern Borders, Jazan, Najran, Al-Baha and Aljouf.

### 2.2. Sample Size

Non-proportional quota sampling (‘soft quotas’) divided by region and gender was used to ensure that respondents were demographically representative of the general population (total number of population: 34,813,871). The sample size was calculated for a β of 0.05 and a power (1 − α) of 0.80; we estimated a minimum sample size of 5004 participants. Due to the limited timeline and the special period of time during conducting this survey, we considered a 10% response rate from the total recruited sample of 50,040. However, since this was an online-based survey capturing public response and attitude, around 200,000 messages have been sent. We aimed to recruit as many participants as possible within the survey period, even after achieving the set sample size.

### 2.3. Recruitment Methods

Because of this exceptional time of the pandemic, household survey was not applicable, thus the study was carried out using phone survey. Based on a list of random mobile phone numbers generated from a governmental database and divided by regions and gender, participants were invited to take part in the study using random digital dialling (RDD). The individuals received a message containing a short description of the study and the survey link. Each user had a unique device identifier linked to the research database, so the user could not submit information more than once. As the data were obtained electronically, no user could submit responses that were missing vital information. Respondents were required to be 16 years or older, living in Saudi Arabia, Arabic speakers and to have heard of COVID-19.

### 2.4. Measurements

Data on socio-demographic characteristics of the respondents, including age, gender, education level, nationality and working status were collected.

The second part of the survey focused on awareness regarding COVID-19 and understanding its risk, requiring true or false responses. These items were developed by the authors and selected according to previous research as well as frequent questions and myths about COVID-19 as reported on the Saudi Ministry of Health (MoH) and WHO websites [11,12,13].

To measure the knowledge among participants, the questionnaire had 12 knowledge questions among different aspects of COVIS-19; 4 regarding clinical presentations, 3 regarding transmission routes and 5 regarding prevention and control of COVID-19. Knowledge score was assessed by multiple choice answers, three possible answers were presented in each question, only one answer was correct. A correct answer was assigned 1 point and an incorrect/unknown answer was assigned 0 points. The total knowledge score ranged from 0 to 12, with a higher score indicating better knowledge of COVID-19 and lower score indicating insufficient/poor knowledge. Cronbach’s α scores were less than 0.6, which prevented us from creating a knowledge scale.

The survey also assessed the level of trust among the public about the government’s ability to handle and control the COVID-19 pandemic. The scale contained 10 questions; 4 items were related to the public’s general perception of the government’s ability to control the disease within the coming months, while the other 6 items specifically asked about their agreement with the implemented measures, such as school closures, travel suspension and mall and restaurant closures. Items measuring trust in authorities were phrased as statements, with response options ranging from strongly agree to strongly disagree (5-point Likert scale). Each answer was scored on a scale from 0 to 4 as following; (strongly agree (4), agree (3), neutral (0), disagree (2), strongly disagree (1). The total score ranged from 0 to 40, with high scores indicating greater agreement that the authorities are to be trusted in handling COVID-19. The Cronbach’s alpha coefficient of the questionnaire was 0.81, indicating high internal consistency.

The survey measured COVID-19’s impact on the public level of anxiety using the Generalized Anxiety Disorder scale (GAD-7). The GAD-7 is the most commonly used diagnostic self-report scales for screening, and severity assessment of anxiety among general population [14]. The scale measures the presence of seven core anxiety symptoms in the last 2 weeks; the total score ranges between 0 and 21 and is split into four categories: normal (0–4), mild (5–9), moderate (10–14) and severe (≥15) anxiety. The reliability and validity of the Arabic version among the Saudi community has also been established [15]. Moreover, participants were asked about their risk perception, concerns of catching COVID-19 and the reasons behind these perceptions. Finally, the questionnaire also measured the respondents’ adoption of protective behaviours, including facemask use, hand hygiene, social distancing, cough etiquette and avoiding social gatherings.

### 2.5. Data Analysis

Participants’ demographics, knowledge and various attitudes and practices were described in numbers and percentages. Knowledge scores according to demographic characteristics were compared with independent-samples *t*-tests and Chi-square tests as appropriate. To identify the factors associated with knowledge, we conducted a multivariable linear regression analysis using all of the demographic variables as independent variables and knowledge and trust in authorities as the outcome variables. We used binary logistic regression analyses to identify the factors associated with the level of anxiety and concern among participants after adjusting for demographics and knowledge using a backward Wald method. We used regression coefficients, standard error and odds ratios (ORs), 95% confidence intervals (CIs) and p values to quantify the associations between variables and study outcomes. The statistical significance level was set at *p* < 0.05 (two-sided). The sample population was weighted to the regions and gender distribution of the Saudi adult population. The weighted and unweighted data change was between 1% and 5%, both results (weighted and unweighted) are presented in all tables. Data analyses were conducted with SPSS version 26.0 and SAS 9.13 statistical software.

### 2.6. Ethics

This study was reviewed and approved by the Human Research Ethics Committee at Saudi Food and Drug Authority (Approval number 2020-002).

## 3. Results

After the end of data collection period, 204,186 participants had been approached, about 90,421 (45.2%) individuals completed the survey. Of those, 68.8% were male, and mean age was 33.6 (SD 8.7, range 16–99) years. Majority (77.5%) had a university-level or higher qualification and 75.7% were employed. The weighted and unweighted characteristics of participants are presented in Table 1.

### 3.1. Knowledge of COVID-19 and Associated Factors

The mean COVID-19 knowledge score was 9.84 (SD: 1.5, range from 0 to 12), and the overall rate of correct answers was 82% (9.84/12 × 100). Over half of participants (56.7%) reported that governmental institutions’ social media accounts were their main source of COVID-19 knowledge, and 84% of them spent up to 3 h a day following COVID-19 pandemic news (Table 2).

Knowledge scores differed significantly across age groups, gender, nationality, education level, employment status and regions (*p* < 0.001). Knowledge scores were higher in regions that reported the first COVID-19 cases (the Eastern region score was 10.03, and the Riyadh region’s score was 9.90) compared to other regions in Saudi Arabia (Table 1).

Multiple linear regression results showed that the age group between 16 and 29 years ([vs. older than 29 Years], β: −0.19, *p* < 0.001), being non-Saudi ([vs. Saudi], β: −0.09, *p* < 0.001), having an education level of high school or less ([vs. bachelor degree or above], β: −0.2, *p* < 0.001), being unemployed/retired ([vs. workers], β: −0.21, *p* < 0.001) and being students ([vs. workers], β: −0.2, *p* < 0.001) were more likely to be associated with poor knowledge (Table 3).

### 3.2. Risk Perception and Anxiety Level

Most participants (69.7%) were either ‘not concerned’ or ‘slightly concerned’ about catching the disease. They gave the following reasons for this: believing that they were under Allah’s (God’s) protection (78.9%), complying with preventive measures (67.1%) and the confidence that they would get the needed health care from the MoH (56%). On the other hand, 30.3% of respondents were highly or moderately concerned about COVID-19 for the following reasons: highly contagious disease (83.2%), high-risk occupation (e.g., being a health care worker ((HCW) or working in crowded places) (56.8%) and having family members or friends who returned recently from countries with a high rate of infection (48%). Table 4 contains more details.

In the multivariate analysis, controlling for participants’ demographics and knowledge level, those who more likely to be concerned about catching COVID-19 were females (OR: 1.78, 95% CI [1.75–1.81], *p* < 0.001), non-Saudis (OR: 1.86, 95% CI [1.82–189], *p* < 0.001), 49 or younger (OR: 1.86, 95% CI [1.75–1.08], *p* < 0.001), had bachelor’s degrees or higher (OR: 1.01, 95% CI [1.02–1.18], *p* < 0.001) and health care workers (OR: 1.16, 95% CI [1.08–1.25], *p* < 0.001) (Table 5).

In total, 49.5% scored 5 or more on the GAD-7 test, indicating anxiety symptoms. Of those, 19.2% scored 10 and above, suggesting moderate to severe anxiety (Table 4). Multivariate analysis showed that females (OR: 1.62, 95% CI [1.59–1.65], *p* < 0.001), non-Saudis (OR: 1.82, 95% CI [1.78–1.87], *p* < 0.001), those 49 and younger (OR: 2.38, 95% CI [2.15–2.64], *p* < 0.001), those holding high school degrees or lower (OR: 1.11, 95% CI [1.01–1.23], *p* = 0.03, health care workers (OR: 1.14, 95% CI [1.05–1.25], *p* < 0.001), those with poor COVID-19 knowledge (OR: 1.15, 95% CI [1.11–1.20], *p* < 0.001) and those concerned about catching the disease (OR; 3.94, 95% CI [3.77–4.12], *p* < 0.001) were more likely to report moderate to high levels of anxiety (Table 5).

### 3.3. Trust in the Governmental Authorities’ Mitigation Plan and Related Factors

When asked about participants level of trust that the COVID-19 pandemic would be successfully controlled globally within the next 2 months, 34.1% of them strongly agreed, 23.2% were neutral and 4.9% strongly disagreed. On the other hand, 53.8% strongly agreed that Saudi Arabia would control the disease successfully within the next 2 months. Moreover, 74.1% of participants were strongly confident that the authorities in Saudi Arabia are undertaking the proper approaches to combat the pandemic.

Regarding the public’s trust and support of interventions implemented by the government to control COVID-19, >78% strongly agree that the implemented plans were essential and will help to control the pandemic. More details are in Table 6.

Overall, the mean trust in the authorities score was 36.04 (SD: 4.4, range from 0 to 40); 16% reported trust scores ≤34, 22% reported trust scores between 35 and 37 and 62% reported scores ≥38.

Multiple linear regression results showed that being a health care worker ([vs. other workers], β: −0.32, *p* < 0.001), having moderate to severe anxiety ([vs. minimal to mild anxiety], β: −0.47, *p* < 0.001) and higher concern levels ([vs. not concerned to slightly concerned], β: −0.02, *p* < 0.001) were significantly associated with low trust in authorities.

### 3.4. Compliance with Personal Preventive Measures

The most commonly practised protective behaviour was hand hygiene—hand washing with water or disinfectants—adopted by 86.9% and 60.3% of respondents, respectively. Facemask use was reported less frequently compared to hand hygiene; 38.5% wore facemasks in public or crowded places and 27.9% used facemasks when developing flu symptoms. On the other hand, the majority of participants practiced social distancing. For instance, 72.5% stayed at home and avoided going out for nonessential business and 49.5% avoided attending social events and family gatherings. Participants’ compliance with the protective measures is summarised in Table 7.

Multivariate analysis showed that males (OR: 1.11, 95% CI [1.07–1.16], *p* < 0.001), non-Saudis (OR: 1.14, 95% CI [1.09–1.19], *p* < 0.001), those concerned with catching the disease (OR: 1.46, 95% CI [1.37–1.55], *p* < 0.001), those with moderate anxiety (OR: 1.23, 95% CI [1.63–1.31], *p* < 0.001), those with severe anxiety (OR: 1.34, 95% CI [1.24–1.44], *p* < 0.001), those reporting higher knowledge scores (OR: 2.09, 95% CI [1.87–2.33], *p* < 0.001), those reporting more trust in authorities (OR: 1.20, 95% CI [1.13–1.27], *p* < 0.001) and younger participants (OR: 1.35, 95% CI [1.27–1.44], *p* < 0.001) were significantly more likely to practice personal protective measures. Moreover, females (OR: 2.93, 95% CI [2.79–3.07], *p* < 0.001), Saudi citizens (OR: 1.24, 95% CI [1.18–1.30], *p* < 0.001), those concerned about catching the disease (OR; 1.06, 95% CI [1.01–1.11], *p* < 0.001), those with higher knowledge scores (OR: 2.77, 95% CI [2.48–3.10], *p* < 0.001), older participants (OR: 1.91, 95% CI [1.76–2.08], *p* < 0.001), those with minimal anxiety (OR: 1.16, 95% CI [1.07–1.25], *p* < 0.001) and those with more trust in authorities (OR: 1.22, 95% CI [1.17–1.27], *p* < 0.001) were significantly more likely to practice social distancing measures (Table 8).

## 4. Discussion

To our knowledge, this is the first Saudi study aiming to understand the public response during the first wave of the COVID-19 pandemic using multiple measurements including knowledge, risk perceptions, anxiety level, trust in authorities and practices. The results obtained in this cross-sectional study showed that knowledge about COVID-19 among Saudis was acceptable, as correct answers from the respondents were 82%. Yet, the total correct answers were lower compared to those from the Chinese public at 90% [11]. Similar results were found during the MERS-CoV outbreak in Saudi Arabia—a low level of knowledge of the disease and its transmission route [9]. Almost all participants answered correctly about COVID-19 transmission routes and hand hygiene effectiveness, yet a lower proportion of correct answers was observed in questions about the disease’s effect on children and the elderly as well as the ability of those with mild cases to transmit the disease to healthy individuals. This may be because COVID-19 is a novel virus, and scientific knowledge about it is still evolving, which may have affected the knowledge levels among the participants. In this study, the knowledge scores were influenced by demographic factors of the participants. Younger age groups, individuals with low levels of education, and foreign residents were associated with poor knowledge of the disease. These results are in agreement with studies conducted during the H1N1 outbreak. A systematic review conducted in 2016 to investigate issues related to communication to the public during the H1N1 pandemic found that older age, higher household income and level of education were positively associated with greater knowledge about H1N [16]. However, in our study, residency status (residents compared to citizens) was associated with lower levels of disease knowledge, and more recent COVID-19 cases in Saudi Arabia have been reported among expatriates than Saudis. Developing communication strategies during pandemics presents a huge challenge for public health agencies in protecting the public from threats. Information about the new risk and its preventive measures needs to be clear and tailored to the population, considering the various socio-demographic determinants, including age, income, education level, ethnicity and residency status. The absence of clear, widespread information may lead to failure in controlling and containing the pandemic.

In this study, the majority of participants reported that governmental bodies’ social media account were the main sources for their information about COVID-19. However, other studies have shown the mainstream media as the primary source of information during the H1N1 pandemic in many countries [17,18,19]. This change can be attributed to social media advancement in recent years and its continued use by government agencies and individuals to spread news and convey public health messages, whereas in the past, news and medical information were spread mainly through mainstream media such as radio and television. Also, the number of people who use social media sites has increased over the years [20]. Nevertheless, to date, there are no studies aiming to evaluate the efficacy of using social media for pandemic awareness and compliance to protective measures compared to other traditional media.

We found that half of the participants were concerned about catching the disease, and this feeling was related to their disease risk perception. In contrast, a low concern of contracting the disease among participants was a result of them not considering themselves at risk and their perception towards the disease. Believing that they are under Allah’s (God’s) protection was found to be the main reason for being unconcerned. Other surveys involving the Saudi public during the MERS-CoV outbreak showed that religious faith led individuals to believe that they are at a lower risk against serious emerging infectious risks [9,21]. Another reported reason was confidence in the government efforts at containing the pandemic and the prevention measures in place, which may have reduced concern about contracting the disease. The results obtained in this study are similar to other studies that have evaluated the response and behaviour of the public during pandemics. For instance, a study by Eastwood et al. (2009) showed that a large percentage of the participants were willing to comply with the government’s directives to reduce the spread of H1N1 in 2009 [22]. In addition, several studies have found that perceived severity and susceptibility were the main driving factors of complying with protective measures among the public. Results from Bults et al. (2011) showed that the uptake of preventive measures by respondents were influenced by their level of risk perception and anxiety [23]. These results are also consistent with those obtained by Steelfisher et al. (2010) and Liao et al. (2009), who indicated that individuals’ perceptions of the guidelines given and the risk of contracting diseases influenced the uptake of the measures [24,25]. Therefore, it is essential for public health agencies to understand public’s perceived risk of emerging diseases to increase their compliance with preventive measures.

Notably, less than half of participants (47.8%) showed anxiety symptoms; 21% of them were struggling from moderate to severe anxiety. The ramifications of mental disorders during pandemics were addressed among Chinese population during this pandemic [26]. The uncertain situation, constant widespread media coverage, lack of specific vaccine or drug and social isolation all contribute to increasing mental burdens, including anxiety and stress. In an effort to limit the risk, the WHO has released information to the public on how to protect their mental health during the COVID-19 pandemic. Moreover, public mental health interventions should be incorporated with other national interventions to control COVID pandemic consequences.

In this cross-sectional study, healthcare workers (compared to other workers) were more likely to be at higher risk of anxiety. Previous studies reported similar results during the 2003 severe acute respiratory syndrome (SARS) outbreak among health care workers [27,28,29]. Protecting health care workers is an important element in containing the COVID-19 epidemic. Health promotion programs with specific interventions should be implemented immediately among health care workers.

The study showed that in terms of symptoms of anxiety and concern, the uptake of preventive measures varied with risk perception and anxiety levels. High anxiety and risk perception led to individuals taking preventive measures, such as avoiding crowded areas and maintenance of high hand hygiene standards. The effect of anxiety on protective behaviours among the public was proven in a study of 10,345 adults, which found that anxiety levels were strongly associated with adoption of protective behaviours during pandemics [8,24,30].

In this study, personal hygiene measures were adopted by a larger number of people than social distancing measures, which would require alterations to their social interactions. While handwashing and use of disinfectants were practiced by 90.4% and 67.1%, respectively, staying at home and avoiding social events and family gatherings were practiced at rates of 76.6% and 55%, respectively. These results are in contrast to the results of a recent study on a Chinese community, where about 90% avoided going to crowded places [11]. Hence, these results may be affected by the fact that the study was conducted before the government adopted semi- and complete curfews. Additionally, in this study, the majority of participants showed positive attitudes and trust in government authorities to control the disease. This may be due to the steady increase of COVID-19 cases in Saudi Arabia compared to other countries and the successful implementation of various controlling measures even before the first case was reported in Saudi Arabia (Figure 1). We found that trust in authorities is strongly associated with the adoption of preventive measures. Therefore, governments should be transparent and clear in communicating with the public, providing up-to-date and culturally appropriate health information. Moreover, engaging in non-traditional methods, such as including public figures, leaders could also help spread public health messages to the most vulnerable populations to increase their knowledge and the adoption of protective measures.

There are some limitations to this study. First, this is a cross sectional study; no follow-up was conducted, and public perception assessed at a point of time may not be representative of consistent beliefs or practice. Second, the data were self-reported and may not reflect the actual behaviour. Also, the sample was selected using random mobile phone numbers that were generated from a government database which excluding households with landing phone; all these could limit the generalisability of the findings. Lastly, there were more males (68.8%) than females and younger age groups were moderately over-represented, but geographically the sample closely reflected the Saudi population distribution. Despite these limitations, this study is the first in-depth analysis of public response during the COVID-19 pandemic and has provided unique data on public perception, anxiety, trust in authorities and practices surrounding COVID-19. Also, because the study used an anonymous online survey, any bias originating from cultural, ethnical or religious factors is eradicated. Additionally, the weighted data by region and gender did not change the outcomes by less than 5% compared to the unweighted data.

## 5. Conclusions

To conclude, this study highlights that Saudi residents are well aware of COVID-19 risk and its transmission routes. This knowledge is influenced by demographic factors of the participants; younger age groups, individuals with low levels of education and foreign residents were associated with poor knowledge of the disease. Moreover, personal protective measures, such as hand hygiene, were more likely to be adopted than social distancing measures. Trust in health authorities, worry, anxiety and levels of knowledge about the disease were related to greater likelihood of implementation of protective practices. Understanding these factors can help public health authorities design culturally appropriate health campaigns to reach the population according to their social-demographic characteristics. Also, our findings recommend that current policy-makers continue providing the public with clear, consistent information, focusing on the practical things that people can do to protect themselves from the disease. Continuous evaluation of public response, knowledge about COVID-19 and the effectiveness of protective measures is essential to better inform policy-makers and identify ways of encouraging behaviour change among public during the early stages of the pandemic.

## Figures and Tables

**Table 1:** Demographic characteristics of participants.

Traits	N (%)	Wt.%	Knowledge Score (Mean, ±SD)	*p* Value
Age Groups				
16–29 Years	24,707 (27)	25.2	9.66 (±1.62)	<0.001
30–49 Years	59,810 (66)	71.5	9.97 (±1.55)	
≥50 Years	5904 (7)	3.3	9.91 (±1.50)	
Gender				
Male	50,617 (56)	68.8	9.86 (±1.6)	0.01
Female	39,619 (44)	31.2	9.83 (±1.4)	
Nationality				
Saudi	69,540 (77)	67.5	9.81 (±1.58)	<0.001
Not Saudi	20,696 (23)	32.5	9.97 (±1.55)	
Education				
≤High School Certificate	27,518 (30.4)	20.8	9.38 (±1.75)	<0.001
Bachelor degree/Diploma	56,657 (63)	77.5	10 (±1.46)	
Postgraduate	5961 (6.6)	1.7	10.4 (±1.33)	
Employment Statue				
Health care worker	6329 (7)	15	10.38 (±1.40)	<0.001
Employed (other than health sector)	38,881 (43)	60.7	9.98 (±1.53)	
Student	12,312 (14)	5.4	9.56 (±1.60)	
Not employed or retired	30,851 (34)	19.4	9.67 (±1.62)	
Region				
Aljouf	542 (0.6)	1.5	9.77 (±1.72)	<0.001
Northern Border	600 (0.6)	1.4	9.64 (±1.77)	
Tabuk	1657 (1.8)	2.8	9.65 (±1.70)	
Hail	808 (0.8)	2.5	9.68 (±1.65)	
Almadenah Almonawara	7186 (8.1)	10.2	9.74 (±1.61)	
AlQasim	2724 (2.7)	4.1	9.74 (±1.61)	
Makkah	31,603 (35.2)	25.2	9.78 (±1.57)	
Riyadh	20,895 (23.2)	23.6	9.90 (±1.56)	
Eastern Region	18,912 (20.1)	16.1	10.03 (±1.52)	
Albaha	463 (0.6)	1.6	9.68 (±1.68)	
Asir	2826 (3.2)	6.2	9.68 (±1.64)	
Jazan	2630 (2.5)	3.1	9.66 (±1.68)	
Najran	537 (0.7)	1.9	9.72 (±1.62)	

**Table 2:** Public knowledge and source of knowledge levels about COVID-19.

Source of Knowledge about COVID-19 *	N (%)	Wt.%
Radio, TV, Magazine	37,976 (42.8)	42.5
Family doctor, GPs	3616 (3.9)	5.1
Friends and family members	28,934 (22.9)	34
Social media twitter and snapchat (personal and news accounts not governmental	40,689 (45)	50.3
Social media twitter and snapchat (Governmental accounts)	60,582 (67.4)	56.7
Ministry of Health website	41,593 (45.5)	40.7
Saudi center of disease control (SCDC)	10,850 (11.6)	9.1
International organisations websites such as WHO CDC	23,509 (25.6)	20.3
How many hours you usually spend daily to in reading COVID-19 news?		
Less than 1 h	44,306 (49.3)	56.5
Between 1 and 3 h	307,435 (34.1)	27.5
Between 3 and 6 h	9042 (9.7)	9.4
More than 6 h	6329 (6.7)	6.5
COVID-19 knowledge (true answer)		
The most common symptoms of COVID-19 are fever, tiredness and dry cough	81,379 (90.2)	87.5
To date, there is no vaccine and no specific medicine to prevent or treat COVID-2019.	69,624 (76.7)	87.3
Everyone must wear facemask when going out	43,402 (47.8)	37.6
COVID-19 can spread from person to person through small droplets from the nose or mouth which are spread from infected person	84,995 (94.1)	92
COVID-19 can spread from contaminated surfaces, then touching eyes, nose or mouth	88,612 (98.6)	91.7
New coronavirus cannot affect children or young adults	69,624 (77.4)	71.3
Older persons and persons with pre-existing medical conditions appear to develop serious illness more often than others	66,911 (74)	63.9
You should avoid direct contact with wild animals and surfaces in contact with animals to protect from COVID 19	58,778 (65.5)	63.4
Isolation and treatment of people who are infected with the COVID-19 virus are the most effective ways to reduce the spread of the virus	88,612 (98.2)	97
People who have come into contact with a person with COVID 19 or arrived from affected countries should be immediately isolated for 14 days	88,974 (98.4)	98.3
Persons with mild symptoms of COVID-2019 or when a fever is not present cannot infect the virus to others	58,773 (64.9)	65.6
Frequent hands hygiene is the most effective way to protect yourself and others against COVID-19	895,165 (99)	98.1
Total mean of knowledge score (total score = 12)	9.84 (SD: 1.5)	

* Multiple answers are allowed.

**Table 3:** Factors associated with low knowledge of COVID-19.

Factor	Coefficient	Standard Error	*p* Value
Student (vs. workers)	−0.191	0.022	<0.001
Unemployed/retired (vs. workers)	−0.215	0.065	<0.001
Age group: 16–29 years (vs. older than 29 Years)	−0.189	0.015	<0.001
Education: high school or less) (vs. bachelor’s degree or above)	−0.0135	0.044	<0.001
Non-Saudi (vs. Saudi)	−0.098	0.015	<0.001

**Table 4:** Public concern and anxiety about COVID-19 pandemic.

Risk Perception	N (%)	Wt.%
Concern of catching COVID 19		
Not concerned	29,386 (32.5)	33.7
A little concerned	32,189 (35.6)	36
Moderately concerned	22,605 (25)	24.6
Very concerned	4701 (5.2)	5.7
Reason for not being concern or little concern		
It is not a fatal disease	14,557 (16.1)	17.9
Because I’m complying with all preventive measures	57,779 (63.9)	67.1
It is only media propaganda	10,307(11.4)	10.3
No cases / very limited number of COVID 19 cases in my city	15,823 (17.5)	16.5
I’m talking care of my health	20,435 (22.6)	24.4
I’m not at risk (not elderly or have chronic condition)	17,541 (19.4)	21.3
I’m under Allah ‘God’ protection	70,076 (77.5)	78.9
If I get disease I’m sure I will get the full health care form MoH	49,550 (54.8)	56
Reason for being moderately concern or very concern		
It is a fatal disease	8106 (31)	33.5
It is a highly contagious disease	78,969 (80.7)	83.2
I’m vulnerable to the disease (working at hospitals or in crowded places)	54,343 (60.1)	56.8
Confirmed cases in my family/friend/work colleague	9657 (10.7)	8.9
Several confirmed cases reported in my city	1058 (1.2)	2.4
I’m at risk (elderly or have chronic condition)	9313 (10.3)	12.1
Since It is a global public health emergency	7414 (8.2)	9.7
I have family member/work colleague arrived from infected countries	41,231 (45.6)	48
Health care system capacity is not able to afford health care if the cases increased sharply	2531 (2.8)	4.3
Anxiety level (GAD 7 scale)		
Minimal anxiety (0–4)	47,199 (52.2)	50.5
Mild anxiety (5–9)	24,232 (26.8)	30.3
Moderate anxiety (10–14)	10,850 (12)	10.6
Severe anxiety (15–21)	8137 (9)	8.6

**Table 5:** Factors associated with concerning and anxiety levels among participants.

Factor	aOR, (95% CI), *p* Value
Factors associated with moderate to very concerning of catching of COVID-19	
Female (vs. Male)	1.78, (1.75–1.81), <0.001
Non-Saudi (Saudi)	1.86, (1.82–189), <0.001
Age 49 years and younger (vs. +50 years)	1.86, (1.75–1.08), <0.001
Health care worker (vs. other workers)	1.16, (1.08–1.25), <0.001
Bachelor degree and higher (vs. below degrees)	1.01, (1.02–1.18), <0.001
Factor associated with moderate to severe anxiety of COVID-19	
Female (vs. Male)	1.62, (1.59–1.65), <0.001
Non-Saudi (vs. Saudi)	1.82, (1.78–1.87), <0.001
Age 49 years and younger (vs. +50 years)	2.38, (2.15–2.64), <0.001
Health care worker (vs. other workers)	1.14, (1.05–1.25), <0.001
High school or below (vs. postgraduate)	1.11, (1.01–1.23), 0.03
Poor knowledge (vs. high knowledge)	1.15, (1.11–1.20), <0.001
Moderate to very concern (vs. no to low concern)	3.94, (3.77–4.12), <0.001

**Table 6:** Trust in the governmental authorities’ mitigation plan.

Level of Confidence of National Plan of Controlling the Disease	N (%)	Wt.%
I’m confident that COVID 19 will be successfully controlled over the world in the next 2 months		
Strongly agree	29,386 (32.5)	34.1
Agree	32,189 (35.6)	33.4
Neutral	22,605 (25)	23.2
Disagree	4701 (5.2)	4.4
Strongly disagree	1265 (1.4)	4.9
I’m confident that COVID 19 will be successfully controlled in Saudi Arabia in the next 2 months		
Strongly agree	50,183 (55.5)	53.8
Agree	26,674 (29.5)	28.5
Neutral	11,302 (12.5)	13.1
Disagree	1537 (1.7)	3
Strongly disagree	542 (0.6)	1.6
I’m confident that Saudi Ministry of Health are taking the proper approaches to control the pandemic		
Strongly agree	69,985 (77.4)	80.2
Agree	16,908 (18.7)	15.8
Neutral	2531 (2.8)	3.1
Disagree	452 (0.5)	0.5
Strongly disagree	180 (0.2)	0.4
I’m confident that all governmental sectors are taking the proper approaches to control the pandemic		
Strongly agree	66,821 (73.9)	74.1
Agree	18,988 (21)	22.5
Neutral	3345 (3.7)	2.1
Disagree	813 (0.9)	0.7
Strongly disagree	217 (0.3)	0.4
School closure decision was essential to control the pandemic		
Strongly agree	51,519 (91.3)	92.1
Agree	4308 (7.6)	6.5
Neutral	421 (0.7)	1.1
Disagree	120 (0.2)	0.2
Strongly disagree	60 (0.1)	0.1
Suspend Umrah entry in Saudi Arabia was essential to control the pandemic		
Strongly agree	80,384 (88.9)	92
Agree	7957 (8.8)	5.2
Neutral	1537 (1.7)	2.1
Disagree	271 (0.3)	0.3
Strongly disagree	180 (0.2)	0.3
Suspend attendance at workplaces in all government and privet agencies for period of (16) days ‘except for health’ was essential to control the pandemic		
Strongly agree	75,501 (83.5)	85.1
Agree	12,478 (13.8)	13
Neutral	1717 (1.9)	1.5
Disagree	361 (0.4)	0.4
Strongly disagree	130 (0.1)	0.1
Mandating self-quarantine for travellers for 14 days was essential to control the pandemic		
Strongly agree	85,086 (94.1)	91.3
Agree	4701 (5.2)	8
Neutral	452 (0.5)	0.5
Disagree	72 (0.08)	0.1
Strongly disagree	45 (0.05)	0.1
Closing of restaurants and shopping malls was essential to control the pandemic		
Strongly agree	50,070 (88.7)	90.5
Agree	5207 (9.2)	7.8
Neutral	842 (1.4)	1.1
Disagree	202 (0.3)	0.5
Strongly disagree	107 (0.1)	0.1
Travel restriction and borders shut down was essential to control the pandemic		
Strongly agree	80,203 (91.1)	89.3
Agree	6600 (7.3)	8.1
Neutral	994 (1.1)	2.3
Disagree	166 (0.2)	0.2
Strongly disagree	71 (0.1)	0.1
Total mean of trust score (total score 40)	36.04 (SD: 4.4)	

**Table 7:** Personal protective measures compliance among public.

Personal Protective Measures	N (%)	Wt.%
Frequent ‘daily’ hand washing with soap when touch things or shaking hand	81,740 (90.4)	86.5
Frequent ‘daily’ hand cleaning with antibacterial gel when touch things or shaking hand	60,672 (67.1)	60.3
Covering mouth and nose while coughing or sneezing	52,715 (58.3)	56.7
Avoidance of touching eyes and mouth	53,890 (59.6)	58.1
Avoidance of hand shaking	54,614 (60.4)	61.5
Wearing facemask in public or crowded places	32,009 (35.5)	38.5
Wearing facemask when developing flu symptoms	27,216 (30.1)	36.4
Wearing facemask while visiting someone with flu symptoms	28,844 (31.9)	27.9
Social distancing measures	N (%)	Wt.%
Avoidance of close contact (kissing, hugging, handshaking) with family members/relatives and friends	48,827 (54)	59.7
Avoidance of social events and family gatherings	49,912 (55.2)	49.5
Stay at home and avoid going out for nonessential business	69,262 (76.6)	72.5
Avoidance of close contact with person who have flu symptoms	49,008 (54.1)	52.4
Frequent cleaning of home surfaces	40,689 (45)	47.2
Frequent cleaning of workplace surfaces	24,142 (26.7)	28.1
None of the above measures	1808 (2)	1.5

**Table 8:** Factors associated with preventive measures compliance.

Factors Associated with Personal Protective Measures Uptake	aOR, (95% CI), *p* Value
Male (vs. Female)	1.11, (1.07–1.16), <0.001
Non-Saudi (vs. Saudi)	1.14, (1.09–1.19), <0.001
Concerning of catching the disease (vs. not concerned)	1.46, (1.37–1.55), <0.001
Moderate anxiety (vs. Minimal anxiety)	1.23, (1.63–1.31), <0.001
Severe anxiety (vs. Minimal anxiety)	1.34, (1.24–1.44), <0.001
High knowledge score (vs. low knowledge)	2.09, (1.87–2.33), <0.001
Age 49 years and younger (vs. +50 years)	1.35, (1.27–1.44), <0.001
Higher score of authorities’ trust (vs. lower score)	1.20, (1.13–1.27), <0.001
**Factors Associated with Social Distancing Measures Uptake**	**aOR, (95% CI), *p* Value**
Female (vs. Male)	2.93, (2.79–3.07), <0.001
Saudi (vs. Non-Saudi)	1.24, (1.18–1.30), <0.001
Concerning of catching the disease (vs. not concerned)	1.06, (1.01–1.11), <0.001
High knowledge score (vs. low knowledge)	2.77, (2.48–3.10), <0.001
Age 30 years and older (vs. 29 years and younger)	1.91, (1.76–2.08), <0.001
Minimal anxiety (vs. severe anxiety)	1.16, (1.07–1.25), <0.001
Higher score of authorities’ trust (vs. lower score)	1.22, (1.17–1.27), <0.001

## Data Availability

Data were available upon request.
